# (Bio)electrochemical ammonia recovery: progress and perspectives

**DOI:** 10.1007/s00253-018-8888-6

**Published:** 2018-03-09

**Authors:** P. Kuntke, T. H. J. A. Sleutels, M. Rodríguez Arredondo, S. Georg, S. G. Barbosa, A. ter Heijne, Hubertus V. M. Hamelers, C. J. N. Buisman

**Affiliations:** 1 Wetsus, European Centre of Excellence for Sustainable Water Technology, Oostergoweg 9, 8911 MA, Leeuwarden, The Netherlands; 2 Sub-Department of Environmental Technology, Wageningen University, Bornse Weilanden 9, P.O. Box 17, 6700 AA Wageningen, The Netherlands; 3 CEB – Centre of Biological Engineering, University of Minho, Campus de Gualtar, 4710–057 Braga, Portugal

**Keywords:** Bioelectrochemical systems, Electrochemical systems, Ammonia recovery, Total ammonia nitrogen, Wastewater treatment

## Abstract

In recent years, (bio)electrochemical systems (B)ES have emerged as an energy efficient alternative for the recovery of TAN (total ammonia nitrogen, including ammonia and ammonium) from wastewater. In these systems, TAN is removed or concentrated from the wastewater under the influence of an electrical current and transported to the cathode. Subsequently, it can be removed or recovered through stripping, chemisorption, or forward osmosis. A crucial parameter that determines the energy required to recover TAN is the load ratio: the ratio between TAN loading and applied current. For electrochemical TAN recovery, an energy input is required, while in bioelectrochemical recovery, electric energy can be recovered together with TAN. Bioelectrochemical recovery relies on the microbial oxidation of COD for the production of electrons, which drives TAN transport. Here, the state-of-the-art of (bio)electrochemical TAN recovery is described, the performance of (B)ES for TAN recovery is analyzed, the potential of different wastewaters for BES-based TAN recovery is evaluated, the microorganisms found on bioanodes that treat wastewater high in TAN are reported, and the toxic effect of the typical conditions in such systems (e.g., high pH, TAN, and salt concentrations) are described. For future application, toxicity effects for electrochemically active bacteria need better understanding, and the technologies need to be demonstrated on larger scale.

## Introduction

Reactive nitrogen is an essential nutrient that feeds many processes on earth (FAO 2015). Large amounts of energy are required in the anthropogenically managed nitrogen cycle to convert inert nitrogen gas (N_2_) from the atmosphere to a variety of reactive nitrogen compounds and back to N_2_ (Maurer et al. 2003). There are ample applications for reactive nitrogen compounds, in the form of NH_3_, NH_4_^+^, NO_3_^−^, NO_2_^−^, and urea, ranging from industrial use to fertilizers. The majority of reactive nitrogen is produced by the Haber-Bosch process which requires an energy input of 37 MJ kg_N_^−1^ and is responsible for about 1 to 2% of the worldwide energy use (Kitano et al. 2012; Strand et al. 2016). After application and use, nitrogen ends up in wastewater both in reactive and nonreactive forms (e.g., proteins) and needs to be removed prior to discharge. The removal of nitrogen in wastewater treatment plants (WWTPs), in which it is converted back into N_2_, requires aeration and therefore contributes significantly to operational costs of a WWTP.

Conventional WWTPs use the two-stage nitrification-denitrification process to remove reactive nitrogen as N_2_. This process requires an energy input of about 45 MJ kg_N_^−1^ removed (Maurer et al. 2003). Anaerobic Ammonia Oxidation (Anammox®) was developed as a more energy-efficient alternative to remove reactive nitrogen as N_2_ (Van Dongen et al. 2001) requiring about 16 MJ kg_N_^−1^ removed (Maurer et al. 2003). The disadvantage of these two processes is that reactive nitrogen is removed as N_2_ rather than recovered as usable reactive nitrogen. Furthermore, N_2_O emissions, a potent greenhouse gas, occur during these nitrogen removal processes (Law et al. 2012).

TAN (total ammonia nitrogen) represents two forms of reactive nitrogen: ammonia (NH_3_) and ammonium (NH_4_^+^). The ratio between both forms is determined by the pH of the solution, and the pKa of the NH_3_/NH_4_^+^ equilibrium is 9.25. For wastewater streams with a high TAN concentration (> 0.5 g L^−1^), such as manure, digestate, urine, black water, landfill leachate, and sludge reject water, TAN recovery is possible by conventional processes. These processes include NH3-stripping, struvite precipitation (i.e., MgNH_4_PO_4_·6H_2_O), and ion exchange (e.g., zeolites), but these are energy intensive and often require chemical dosing (Maurer et al. 2006).

## (Bio)electrochemical systems for TAN removal

**Fig. 1:**
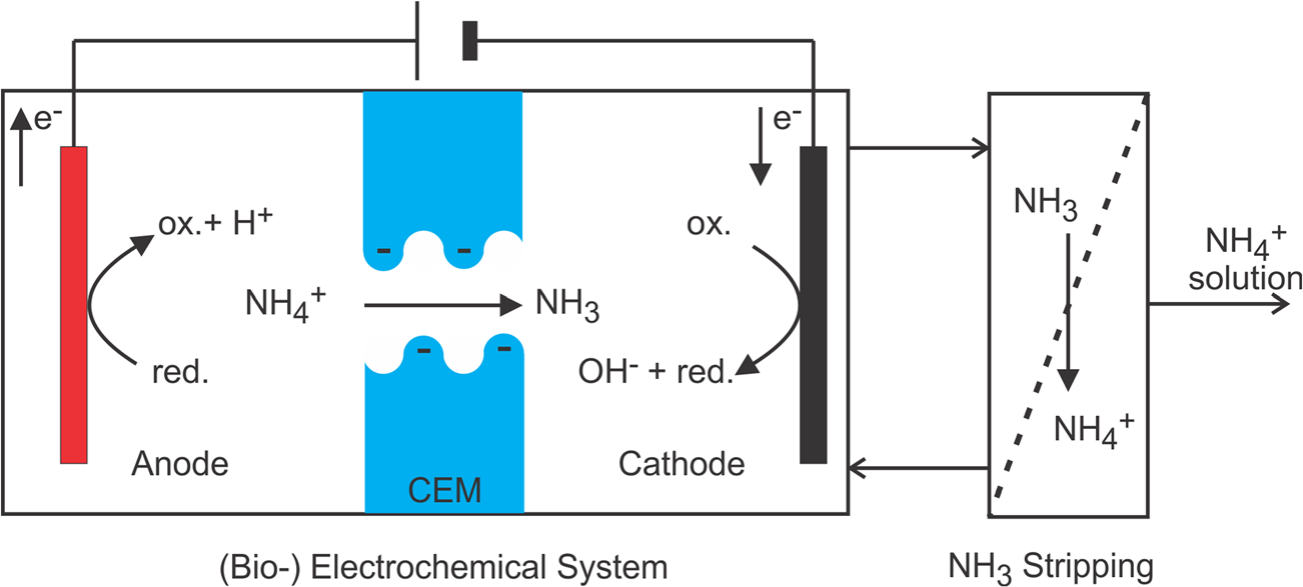
Scheme of a (B)ES for TAN recovery. The coupling of the anodic oxidation reaction with the cathodic reduction reaction induces an electric current across the electric circuit. This electron transport is matched by cation transport over the cation exchange membrane separating anode from cathode compartment to maintain electroneutrality. Therefore, ammonium and other cations are concentrated in the cathode compartment. On the right, an additional process step can be seen, for example stripping, which can be included to extract and recover the TAN in a concentrated form

In recent years, TAN recovery from wastewater by electrochemical systems (ES) and bioelectrochemical systems (BES) has been investigated as an alternative to the conversion of TAN to N_2_ via nitrite or nitrate (Kelly and He 2014; Ledezma et al. 2015; Rodríguez Arredondo et al. 2015). Figure 1 shows a scheme of TAN recovery from wastewater using ES or BES, coupled with a recovery unit (e.g., NH_3_ stripping). TAN recovery in BES relies on electrical current; the flow of electrons (negative charge) is the driving force for the transport of positively charged ammonium ions. When a cation exchange membrane (CEM) is used, ammonium is separated from the feed solution. The source of electrons is an oxidation reaction at the anode, and the sink of electrons is a reduction reaction at the cathode.

**Fig. 2:**
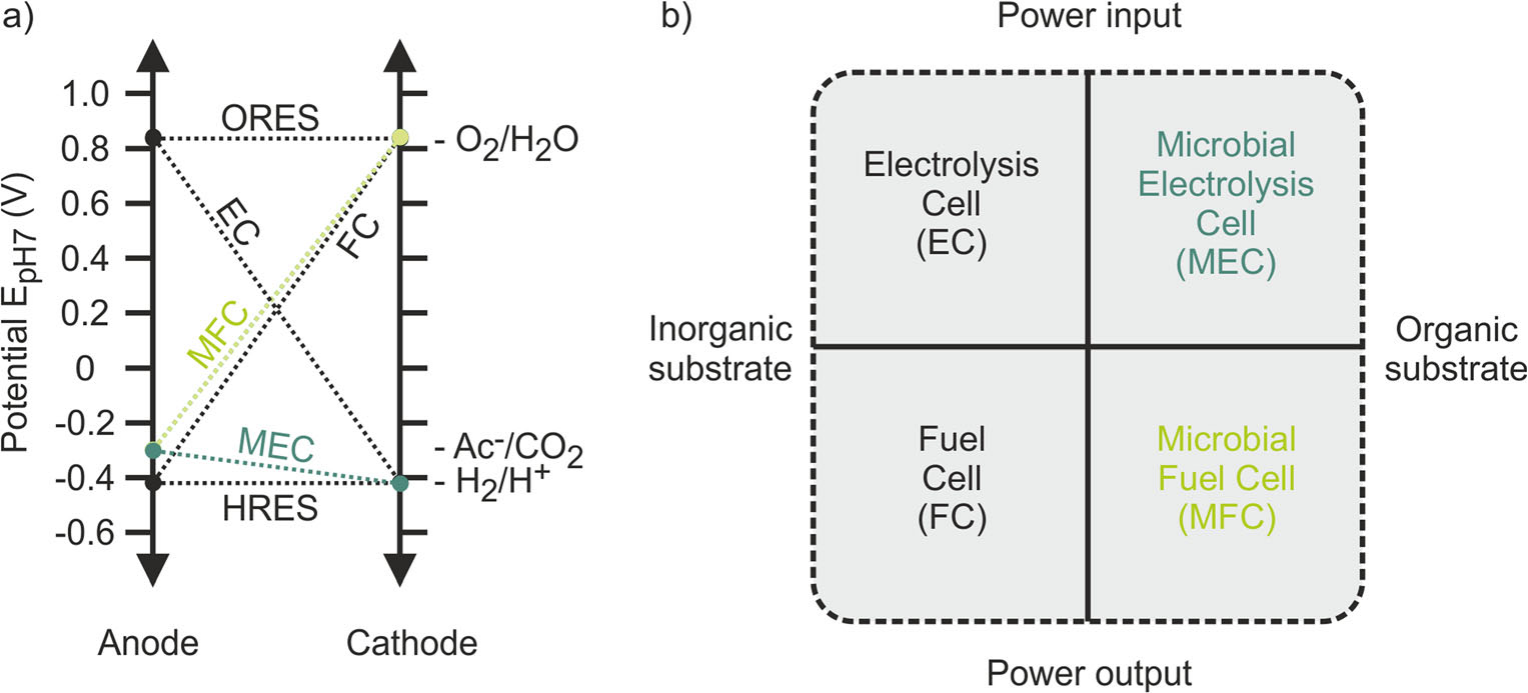
**a** Representations of anode and cathode potential (E_pH7_) in (bio)electrochemical systems. The conditional potentials were determined using the Nernst equation assuming a temperature of 25 °C, a partial pressure of 1 atm of the respective gasses in the headspace, a pH of 7 at the anode and cathode and an acetate (Ac^−^) and bicarbonate (HCO_3_^−^) concentration of 5 mM. All potentials are reported versus normal hydrogen electrode (NHE). While a positive slope indicates that power is produced during (B)ES operation, a negative slope indicates power is consumed during (B)ES operation. A horizontal line indicates that theoretically no additional energy input is required. ORES oxygen recycling electrochemical system, HRES hydrogen recycling electrochemical system, EC electrolysis cell, FC fuel cell, MFC microbial fuel cell, MEC microbial electrolysis cell. **b** Classification of (bio)electrochemical system used for TAN recovery according to power production or consumption and use of organic or inorganic substrates. Both ORES and HRES can be included under EC or MEC classification, depending on their anodic catalyst

In case of a BES, the anodic oxidation reaction is catalyzed by microorganisms, whereas in an ES, purely electrochemical reactions take place. Depending on the counter reaction at the cathode, either energy can be harvested, or energy needs to be invested to drive these reactions. Figure 2 gives an overview of the various (B)ESs that have been used for TAN recovery. These systems can be divided in systems that produce electricity, i.e., fuel cells, and systems that need electrical energy input to drive the reactions, i.e., electrolysis cells. Additionally, these systems can be divided according to the reaction at the anode. BESs make use of microorganisms that catalyze the oxidation of organic matter (COD) into electrons, protons, and bicarbonate. In electrochemical systems, usually inorganic substrates are oxidized, e.g., water can be oxidized to oxygen, protons, and electrons, or hydrogen can be oxidized to protons and electrons. In these electrochemical oxidation reactions, commonly, noble metal catalysts are used, although hydrogen oxidation can also be catalyzed by microorganisms (Ntagia et al. 2016; Rodenas et al. 2017).

Table 1 shows an overview of the possible electrode reactions which can be used in (B)ESs for TAN recovery. The combination of one oxidation and one reduction reaction defines the type of (B)ES for TAN recovery (Fig. 2a). Figure 2b shows a classification of the four types of (B)ESs according to the type of substrate utilized (inorganic vs. organic) and the resulting net power input or output.

**Table 1:** Overview of standard potentials (E^0^) (Lide 1994) and conditional potentials (E_pH7_) of the electrode reactions used in (bio)electrochemical systems for TAN recovery. Conditional potentials were determined using the Nernst equation assuming a temperature of 25 °C, a partial pressure of 1 atm of the respective gasses in the headspace, a pH of 7 at the anode and cathode, and an acetate (CH_3_COO^−^) and bicarbonate (HCO_3_^−^) concentration of 5 mM. All potentials are reported versus normal hydrogen electrode (NHE)

Electrode		Reaction	E^0^ (V vs NHE)	E_pH7_ (V vs NHE)
Cathode	Hydrogen evolution	2H_2_O + 2e^−^ → H_2_ + 2OH^−^	− 0.828	− 0.414
	Oxygen reduction	O_2_ + 4e^−^ + 2H_2_O → 4OH^−^	0.401	0.815
Anode	Acetate oxidation	2HCO_3_^−^ + 9H^+^ 8e^−^ → CH_3_COO^−^ + 4H_2_O	0.187	− 0.296
	Oxygen evolution	O_2_ + 4H^+^ + 4e^−^ → 2H_2_O	1.229	0.815
	Hydrogen oxidation	2H^+^ + 2e^−^ → H_2_	0	− 0.414

In this manuscript, the different types of (B)ESs and TAN recovery techniques described in literature are reviewed. Furthermore, performance of (B)ESs in terms of COD removal and TAN recovery are summarized and wastewater streams most suitable for these applications based on the (biodegradable) COD to TAN ratio are described. Finally, an overview of the microorganisms found in TAN recovering BESs is given, an insight in the limitations that come with using wastewaters high in TAN is given and future directions for research and development are discussed.

## TAN recovery from wastewater

The advantage of TAN recovery by (B)ESs compared to conventional TAN recovery methods is that the electrical current aids in the TAN recovery, since it allows to concentrate the TAN in the cathode compartment prior to recovery and minimizes the chemical dosing requirements (Rodríguez Arredondo et al. 2015). The combination of both factors allows for more energy efficient TAN recovery compared to conventional recovery methods.

As described, most (bio)electrochemical systems for TAN recovery rely on CEMs to transport and concentrate TAN from the feed stream using electric current as the driving force. While the concentrated TAN-rich stream obtained at the cathode could be considered the final product, without further extraction, several methods have been investigated to recover TAN as a more refined or pure product. Table 2 summarizes the performance of BES employing different TAN recovery methods: stripping, transmembrane chemisorption (TMCS), forward osmosis, and concentration, which will be discussed in more detail here.

**Table 2:** Performance of electrochemical (EC and HRES) and bioelectrochemical systems (MFC and MEC) for TAN removal or recovery reported in recent literature; the mode of operation (mode), i.e., continuous (c) or batch (b) operation; the load ratio (L_N_); the current densities (j, A m^−2^) obtained; the TAN removal rate (rate, g_N_ m^−2^ day^−1^); the TAN transport efficiency over CEM (η_N_ = %); and the electric energy demand (Energy, kWhkg_N_^−1^)

	Wastewater	Type	Mode	Recovery method	j A m^−2^	L_N_	Recovery/removal %	Rate, g_N_ m^−2^ day^−1^	η_N_ %	Energy, kWh kg_N_^−1^	Reference
ES	Urine (after P recovery)	HRES	c	TMCS	10	1.3	82.00	78	57	8.5	Kuntke et al. (2017)
	Urine (after P recovery)	HRES	c	TMCS	20	1.2	73.00	151	58	7.3	Kuntke et al. (2017)
	urine (after P recovery)	HRES	c	TMCS	50	1.3	73.00	342	55	15.6	Kuntke et al. (2017)
	Urine (after P recovery)	EC	c	TMCS	20	2.72	89.00	82	33	18.0	Rodríguez Arredondo et al. (2017)
	Urine (after P recovery)	EC	c	TMCS	50	6.5	92.00	89	13	46.3	Rodríguez Arredondo et al. (2017)
	Urine (after P recovery)	EC	c	TMCS	50	1.18	63.00	335	53	13.6	Rodríguez Arredondo et al. (2017)
	Digestate (synthetic)	EC	c	Stripping	30	0.96^a^	41.00	142	38	16.8 ± 1.4	Desloover et al. (2012)
	Digestate (synthetic)	EC	c	Stripping	10	0.01^a^	1 ± 0	120	96	5 ± 0.1	Desloover et al. (2012)
	Digestate	EC	c	Stripping	10	0.8^a^	38 ± 2	51	41	13.1 ± 0.9	Desloover et al. (2012)
	Digestate	EC	c	Stripping	20	1.6^a^	58 ± 3	90	36	16.7 ± 0.9	Desloover et al. (2012)
	Digestate	EC	c	Stripping	30	2.4^a^	63 ± 1	94	25	26.0 ± 0.7	Desloover et al. (2012)
	Urine (synthetic)	EC	c	Stripping	30	0.74^a^	53 ± 1.0	253	67	9.5	Luther et al. (2015)
	Urine (synthetic)	EC	c	Stripping	50	1.23^a^	80.7 ± 1.6	384	61	12.4 ± 0.4	Luther et al. (2015)
	Urine	EC	c	Stripping	40	1.34^a^	75.0 ± 0.5	235	58	14.7	Luther et al. (2015)
	Synthetic	EC	c	Stripping	30	0.96^a^	41 ± 2	143	38	16.8 ± 1.4	Gildemyn et al. (2015)
	Urine	EC	c	Stripping	20	0.5^a^	86.50	n.a.	n.a.	2.9^a^	Christiaens et al. (2017)
	Urine	EC	c	Stripping	20	0.5^a^	68.4 ± 14	n.a.	n.a.	3.9^a^	Christiaens et al. (2017)
BES	Urine (after P recovery)	MFC	b	Stripping	2.6	0.06^a^	1.6^a^	9.56	29^a^	-2.8^a^	Kuntke (2013)
	Reject water	(M)EC	b	Stripping	28.2^a^	n.d.	79.0	n.a.	n.a.	20.5	Wu and Modin (2013)
	Urine (after P recovery)	MEC	c	Concentration	14.6	0.39^a^	33.40	162	89	2.3	Kuntke et al. (2014)
	Urine (after P recovery)	MEC	c	TMCS	1.6	0.61^a^	46.00	19	69	2.6	Kuntke et al. (2016)
	Urine (after P recovery)	MEC	c	TMCS	1.6	0.26	26.50	27	96	1.1	Zamora et al. (2017)
	Synthetic	MEC	c	Stripping	27	0.84^a^	51 ± 0.5	226	67	6.04 ± 1.78	Gildemyn et al. (2015)
	Urine (synthetic)	MEC	c	Concentration	29.3	0.42^a^	49.5 ± 1.8	519.5	141	2.38	Ledezma et al. (2017)
	Digestate (synthetic)	MFC	b	Stripping	7.6	0.84^a^	88.0	80	119	−0.1	Zhang and Angelidaki (2015a)
	Digestate (synthetic)	MFC	c	Stripping	4.3	0.30^a^	51.67^a^	86	n.r.	0.03^a^	Zhang and Angelidaki (2015b)
	Synthetic wastewater	MEC	b	Stripping	2.7	n.d.	n.r.	11.8	n.r.	2.67	Zhang and Angelidaki (2015c)
	Pig slurry	MFC	c	Stripping	0.07	n.d.	n.r.	3.7	n.r.	n.r.	Sotres et al. (2015)
	Pig slurry	MEC	c	Stripping	n.r.	n.d.	n.r.	25.5	n.r.	n.r	Sotres et al. (2015)
	Synthetic (lifestock) wastewater	MEC-O_2_	b	Stripping/FO	1.8	n.d.	81.00	7.6	49	5.1	Qin and He (2014)
	Landfill leachate	MEC	b	Stripping/FO	0.76	n.d.	63.7 ± 6.6	n.r.	n.r.	5.5^a^	Qin et al. (2016)
	Synthetic (lifestock) wastewater	MFC-O_2_	c	FO	2.6	0.7^a^	52.5 ± 4.7	25.9^a^	79.5^a^	n.r.	Qin et al. (2017)
	Synthetic (lifestock) wastewater	MEC	b	Stripping/FO/MAP	0.76^a^	n.d.	99.7 ± 13	n.r.	n.r.	1.1 ± 0.05	Zou et al. (2017)

*n.a.* not applicable, *n.d.* not determined (i.e., too little information provided to calculate), *n.r.* not reported

^a^Calculated from provided data

### Concentration

Using a (B)ES for the removal of TAN from the anode (feed) solution and its transport to the cathode (concentrated) solution results in separation of the TAN from the wastewater stream and a concentrated TAN solution in the cathode (Kuntke et al. 2011). The low transport rates obtained in MFCs can be increased by changing them into MECs, which produce more current (maximum of 23 compared to 0.5 A m^−2^) due to the applied power (Kuntke et al. 2014). These early studies generally resulted in low TAN recoveries. The main limitations were the build-up of a TAN concentration gradient across the CEM resulting in ammonia transport from cathode to anode and low current densities that are inherent to the bioanode.

Recently, a modification of this recovery concept was evaluated using an MEC consisting of three compartments; anode, cathode, and an additional compartment in between for the concentration or recovery of the product (Sleutels et al. 2010; Ledezma et al. 2017). The so-called “bio electroconcentration process” relies on the TAN transport through a CEM from the feed solution in the anode to the concentrate compartment. Afterwards, the TAN depleted feed solution was passed over an aeration column to remove excess COD aerobically and then fed to the cathode. The (bi)carbonate ion was transported through an AEM from the cathode to the same concentrate compartment, thereby achieving a concentration of ammonium and bicarbonate along with other ions. Ammonium bicarbonate salt was recovered after energy intensive freezing (Ledezma et al. 2017).

### Stripping

Stripping is the commonly used technique to remove ammonia from concentrated wastewater. Stripping is achieved by sparging a highly dispersed gas through a TAN-containing solution. The high solubility of ammonia requires high gas flow rates, an elevated temperature and high pH for a complete TAN recovery, resulting in a high energy demand (90 MJ kg_N_^−1^) for conventional stripping (Maurer et al. 2003; Maurer et al. 2006; Pradhan et al. 2017).

Several (B)ESs were studied with an integrated ammonia stripping process at the cathode, showing its potential to reduce the energy demand for TAN recovery compared to conventional stripping alone. In these (B)ESs, TAN from feed or anode solution was concentrated in the cathode (concentrate) compartment and recovered in an acid solution via ammonia stripping. While earlier attempts with BESs showed limited TAN recovery and the need for more effective stripping devices (Kuntke et al. 2012; Wu and Modin 2013), experiments with ES in combination with stripping showed high TAN removal rates and high TAN recoveries (Desloover et al. 2012; Gildemyn et al. 2015; Luther et al. 2015). In more recent work, the successful integration of cathodic ammonia stripping for TAN recovery in BES was shown (Gildemyn et al. 2015; Zhang and Angelidaki 2015a; Zhang and Angelidaki 2015b; Sotres et al. 2015; Zhang and Angelidaki 2015c). Recent work from Christiaens et al. (2017) demonstrated the concept of microbial protein production from TAN recovered in an ES ammonia stripping system, in which protein is produced as a higher value product compared to fertilizers (e.g., (NH_4_)_2_SO_4_, (NH_4_)HCO_3_,) recovery (Christiaens et al. 2017).

### Transmembrane chemisorption

TAN can also be recovered from solution using of a membrane contactor in a process called transmembrane chemisorption (TMCS) or (ammonia) membrane stripping. These membrane contactors employ microporous gas-permeable hydrophobic membranes (e.g., PTFE- or PP-based membranes). The driving force for TAN recovery is the ammonia concentration gradient across the membrane, which requires an elevated pH (> 8.5) in the feed solution and an acidic pH (< 7) on the product side (Ahn et al. 2011; Ulbricht et al. 2013; Garcia-González and Vanotti 2015). Membrane stripping or TMCS was first integrated with BES for the TAN recovery from the cathode and to recycle proton shuttles (NH_3_ and CO_2_) between anode and cathode liquid to enhance BES performance (Sleutels et al. 2016b; Kuntke et al. 2016). Afterwards, TAN recovery in a scaled-up MEC (0.5 m^2^) using TMCS was successfully demonstrated, showing the potential for an energy-efficient nutrient recovery system (Zamora et al. 2017; Igos et al. 2017). The integration of TMCS with an ES and especially in a hydrogen recycling electrochemical system (HRES) showed that TAN recovery can be achieved at higher rates and with comparable energy input to BES (Kuntke et al. 2017; Rodríguez Arredondo et al. 2017). The integration of TMCS within (B)ES is less complex and therefore more robust than the integration of ammonia stripping with (B)ES (Kuntke et al. 2016).

### Forward osmosis

Forward osmosis (FO) is a process in which water is separated from dissolved solutes, using a semipermeable membrane. This process is based on an osmotic pressure difference between two solutions due to a concentration difference. The osmotic pressure difference causes a flow of water from the feed solution to the concentrate (draw) solution, thereby extracting water (Cath et al. 2006). FO can be used to aid TAN removal from wastewater by increasing the TAN content (Qin and He 2014; Lu et al. 2015; Qin et al. 2016). The TAN-containing wastewater is first supplied to the BES anode, and TAN is transported to the cathode and subsequently recovered as NH_4_HCO_3_ using an ammonia stripping process. The produced NH_4_HCO_3_ can be used in the FO processes step as the draw solution to concentrate the TAN-depleted effluent of the BES anode. Afterwards, this concentrated wastewater can be mixed with fresh wastewater and supplied as feed for the BES anode. This process has also been integrated with an additional struvite (MgNH_4_PO_4_·6H_2_O, MAP) precipitation step to maximize nutrient recovery, increasing the complexity of the treatment process (Zou et al. 2017). As an alternative, direct integration of the FO membrane in the BES, as a separator between anode and cathode compartment, showed promising results and simplified the overall system (Qin et al. 2017).

## Assessing the performance of (B)ES for TAN recovery: the importance of load ratio

The performance of (bio)electrochemical systems for TAN recovery can be characterized by several parameters with relation to TAN recovery: (i) removal efficiency, (ii) recovery efficiency, (iii) removal rate, and (iv) specific energy input.

The removal efficiency (%) describes the part of TAN that is removed from the influent, while the recovery efficiency (%) describes the part of TAN in the influent that is recovered as a final product. The TAN removal rate (g_N_ m^−2^ day^−1^) describes the rate at which TAN is removed from the influent with respect to the membrane area of the (B)ES. Finally, the specific energy input (kWh kg_N_^−1^) is the energy required to recover or remove TAN. It is challenging to compare the performance of (bio)electrochemical system solely based on the parameters just described, due to the fact that each system is operated with a specific intention. For example, the aim may be to obtain a high rate (current density, removal rates), a high removal and recovery efficiency, or minimal energy input or maximal energy gain. These aims cannot all be achieved at the same time, as there are trade-offs among them. For example, ECs operating at high rate normally require higher energy input than systems operating at low rate. Furthermore, MFCs in comparison to MECs produce electric energy, but operate at much lower rates. To enable better comparison between studies and to understand the relationship between current and TAN transport and recovery, the load ratio (L_N_) model was developed. L_N_ (unitless number) relates the current density of an (B)ES to the TAN loading (Rodríguez Arredondo et al. 2017).

1 $$ {L}_N=\frac{j}{C_{\mathrm{anolyte},\mathrm{TAN}}\kern0.5em {Q}_{\mathrm{anode}}\kern0.5em \frac{F}{A_m}} $$

where *j* is the current density (A m^−2^), *C*_anolyte, TAN_ is the molar concentration of TAN in the anolyte inflow (mol m^−3^), *Q*_anode_ is the anolyte inflow rate (m^3^ s^−1^), *F* the Faraday constant (96485 C mol^−1^), and *A*_*m*_ is the surface area of the cation exchange membrane (m^2^).

This L_N_ is a useful parameter to assess under which conditions a system should optimally be operated in terms of nitrogen removal efficiency and energy input. The L_N_ is defined as the ratio between the current that is produced or applied to the system and the TAN loading to the system, expressed as current. A L_N_ equal to 1 thus describes a situation in which the current (applied or produced) matches the TAN loading. For a L_N_ higher than 1, there is an excess of current compared to the TAN loading, whereas a L_N_ lower than 1 means that there is not enough current to transport all the TAN. It was found that, for ES treating human urine, TAN recovery increases with increasing L_N_, and optimum TAN recovery was reported for an L_N_ of approximately 1.3.

**Fig. 3:**
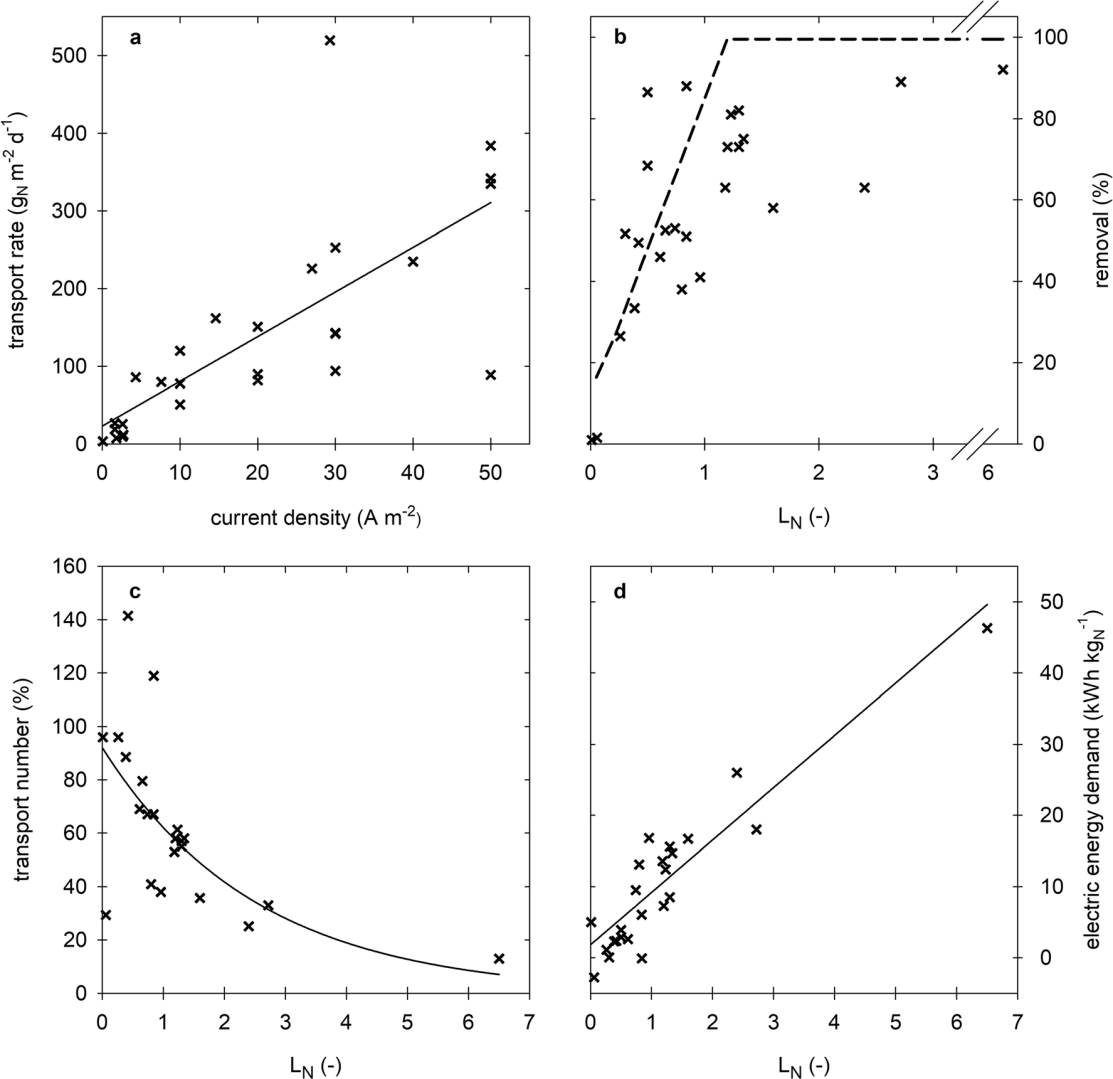
**a** Relation between current density and TAN removal rate including a linear regression to illustrate the trend. **b** Relation of load ratio (L_N_) and TAN recovery/removal including the L_N_ model (dashed line) (Rodríguez Arredondo et al. 2017). **c** Relation between L_N_ and transport efficiency over the CEM including a nonlinear regression to illustrate the trend. **d** Relation between L_N_ and energy demand including a linear regression to illustrate the trend

To see if this is also valid for other studies the performance of different (B)ES for TAN recovery reported in literature is summarized (Table 2), which are graphically represented in Fig. 3. First, Fig. 3a shows that TAN transport rates increase more or less linearly with increasing current density which is in line with expectations as current is the main driving force for ammonium transport. This is only valid, however, when sufficient TAN is available in relation to the current applied or generated. Therefore, the L_N_ is a good tool to compare TAN removal (Fig. 3b), TAN transport numbers (Fig. 3c), and specific energy input (Fig. 3d) in different studies.

Figure 3b shows the relation between L_N_ and removal efficiency. In general, the nitrogen removal efficiency is expected to follow an increasing trend with L_N_, reaching a maximum at a specific L_N_. Depending on the system design, wastewater composition, and operational conditions, this maximum will be different. With the exception of three data points (which corresponds to the only study performed in batch and where TAN containing wastewater is directly supplied to the cathode), the removal efficiencies of different (bio)electrochemical systems were lower than 60% for an L_N_ < 1. For an L_N_ between 1 and 1.3, the recovery efficiencies were between 63 and 82%. The two data points at an L_N_ higher than 1.3 show recovery efficiencies around 60%. Whereas in theory, at an L_N_ > 1.3, 100% removal efficiency should be feasible, in practice, lower removal efficiencies are observed. These lower efficiencies are most likely due to complications during the experiments, such as low stripping-absorption efficiency, or due to lower TAN concentrations in the wastewater compared to the other studies (Desloover et al. 2012).

Figure 3c shows the relation between the L_N_ and the TAN transport number over the CEM. The TAN transport number shows which part of the current is used for TAN transport and thus shows if TAN or other cations like K^+^ and Na^+^ are transported. When current is low compared to TAN loading (low L_N_), most of the charge is transported through NH_4_^+^; when current is high compared to TAN loading (high L_N_), a decrease in TAN transport number can be observed, meaning that transport of other cations becomes more important.

Finally, Fig. 3d shows that the energy input increases with increasing L_N_. The data points with an energy input lower than zero correspond to microbial fuel cells, in which electricity is harvested. Although there is a trend of increasing energy input with increasing L_N_, it is clear that there are some exceptions. For example, in the study of Kuntke et al. (2017), the energy input of three experiments is very different even though the L_N_ is the same. The reason for these differences originates from differences in overpotentials (for both anode and cathode) and differences in transport losses over the CEM at the applied current densities (Kuntke et al. 2017).

Although L_N_ is a useful parameter to compare different studies, the exceptions or outliers show that making comparisons by means of the L_N_ has its limitations. There are other factors that also play a role in the removal or recovery of nitrogen in current-driven systems that are not taken into account in the L_N_ concept, such as wastewater composition. Also, when results are obtained before steady-state conditions are reached, not only for current and potentials, but also for electrolyte compositions (pH, conductivity), they may show different behavior regarding ion transport compared to steady-state results (Sleutels et al. 2013).

## Potential wastewaters streams for ammonia recovery in (B)ES

The L_N_ can be used to determine the minimum current required to remove a certain fraction of the TAN from a certain wastewater stream (Fig. 3b). However, one additional limitation specifically relevant for BESs is the concentration of biodegradable organic material in the wastewater, which affects the current that can be produced at the bioanode. The COD/TAN ratio of wastewaters is a given value, and thus, the question arises if the wastewater under consideration contains sufficient COD to recover all TAN.

**Fig. 4:**
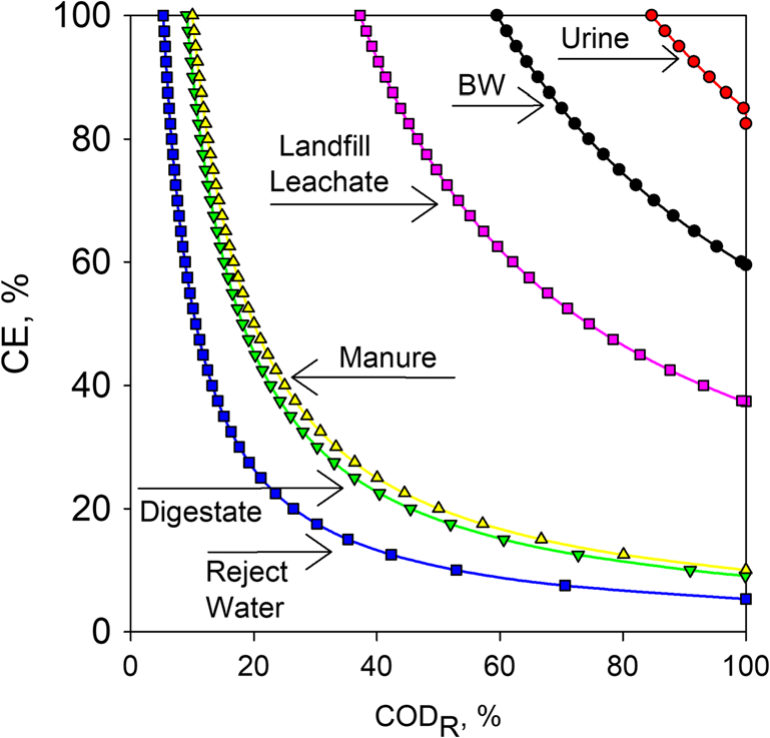
Required minimum CE and COD_R_ of selected wastewater to reach a recovery potential (RP) value of 1. Wastewaters selected were as follows: source separated urine after struvite recovery (Zamora et al. 2017), effluent of a black water (BW) UASB (de Graaff et al. 2010), digestate (Desloover et al. 2012), swine manure (Hernández et al. 2011), municipal wastewater digestate supernatant (reject water) (Henze et al. 2008), and landfill leachate (El-Gohary and Kamel 2016). The calculations are based on Eq. 2 using reported literature values for TAN and COD. A TAN transport efficiency (*η*_*N*_) of 60% was chosen based on a load ratio of approximately 1.2 (Table 2, Fig. 3c). RP values above 1 indicate that sufficient oxidizable organic matter is available to recover all TAN

In BES, the maximum number of electrons available from the substrate depends on the COD removal (COD_R_) and the Coulombic efficiency (CE). CE is of importance, since electrons may be transferred to alternative terminal electron acceptors (e.g., methane, oxygen, metals, sulfate) in competing processes and do not end up at the anode (Sleutels et al. 2011; Sleutels et al. 2016a). Therefore, the suitability of a wastewater for TAN recovery by BES can be assessed by evaluating the number of moles of electrons available for the anodic oxidation in relation to the moles of TAN present in the wastewater. This recovery potential (RP) can be calculated according to

2 $$ RP=\frac{z\kern0.5em \left[\mathrm{COD}\right]\kern0.5em \mathrm{CE}\kern0.5em {\mathrm{COD}}_r\kern0.5em {\eta}_N}{\left[\mathrm{TAN}\right]} $$

where *z* is the amount of electrons transferred during the oxidation (4), [COD] is the COD concentration (mol L^−1^), CE is the Coulombic efficiency (%), COD_*r*_ is the COD removal efficiency (%), [TAN] is the TAN concentration (mol L^−1^), and *η*_*N*_ is the TAN transport number (%). A recovery potential greater than 1 means sufficient degradable COD is available to recover all TAN, while a recovery potential smaller than 1 indicates that only part of the TAN can be recovered. Figure 4 shows the minimum COD removal efficiency and Coulombic efficiency required during TAN recovery by a BES system to reach a recovery potential of 1 for six wastewaters streams (based on a *η*_*N*_ of 60% and an L_N_ of ~ 1.2, see Table 2). These wastewater streams were selected because of their high COD and TAN concentrations; source separated urine after struvite recovery contains a COD concentration of 4.5 g L^−1^ and a TAN concentration of 4 g L^−1^ (Zamora et al. 2017); effluent of a black water (BW) UASB contains 2.4 g COD L^−1^ and 1.5 g TAN L^−1^ (de Graaff et al. 2010); digestate contains 22 g COD L^−1^ and 2.1 g TAN L^−1^ (Desloover et al. 2012); swine manure contains 29.5 g COD L^−1^ and 3.1 g TAN L^−1^ (Hernández et al. 2011), municipal wastewater digestate supernatant (reject water) contains 9 g COD L^−1^ and 0.5 g TAN L^−1^ (Henze et al. 2008); and landfill leachate contains 13.3 g COD L^−1^ and 5.2 g TAN L^−1^ (El-Gohary and Kamel 2016).

The most suitable wastewater based on the evaluation in Fig. 4 is reject water followed by digestate, swine manure, landfill leachate, UASB BW effluent, and urine. This order also corresponds to the COD to TAN ratio of these wastewater, which shows that the higher COD/TAN ratio, the lower the CE and COD_*r*_ can be to theoretically recover all TAN. Another important aspect to consider is that the efficiency of a BES treatment is dependent on the biological activity. This activity may be considerably affected if the wastewater is contaminated with toxic or recalcitrant compounds, or only part of the COD is available for biological degradation. Many industrial wastewaters as well as effluent streams from digesters have little to no biodegradable organic compounds present, where most of the biodegradable COD has already been converted into methane. In case too little biodegradable COD is available to recover all TAN, additional electron donor can be provided, for example by addition of extra biodegradable organic matter or through hydrogen gas recycling from the cathode to the bioanode (Ntagia et al. 2016; Rodenas et al. 2017; Kuntke et al. 2017).

## Biological aspects: microorganisms

In BESs, microorganisms act as a catalyst for the removal of COD in the anode and are therefore primarily responsible for the conversion of COD into electrons. These microorganisms that interact with the anode are referred to as electrochemically active bacteria (EAB). Mixed cultures in BES do not only consist of EAB, but also contain fermentative bacteria, which convert complex organic compounds into smaller metabolites and are not electrochemically active. These metabolites may then be used as substrate by the EAB (Freguia et al. 2008; Parameswaran et al. 2009). In general, EAB grow close to the electrode surface, forming a biofilm, while the fermentative bacteria dominate the top of the biofilm (Moscoviz et al. 2016).

Generally, mixed cultures on bioanodes are mainly enriched in bacteria assigned to the *Proteobacteria* and *Firmicutes* phyla (Rabaey et al. 2004; Cerrillo et al. 2016; Hari et al. 2016; Lu et al. 2017). However, the predominance of these bacteria on bioanodes is greatly influenced by the environmental conditions. In addition to factors such as inoculum (Ishii et al. 2017), organic load (Cetinkaya et al. 2017), and temperature (Larrosa-Guerrero et al. 2010), the microbial community composition is highly affected by the substrate type, which subsequently influences BES performance (Chae et al. 2009). The effect of the anode potential on the microbial community is under debate (Croese et al. 2013; Dennis et al. 2016).

Here, studies reporting the microbial communities found in BESs working with high concentrations of TAN will shortly be addressed. These communities can be of special interest since they might be tolerant to high concentrations of TAN and are capable of degrading complex forms of COD.

Sotres and co-workers evaluated the microbial community dynamics in a two-chambered MFC fed with the liquid fraction of pig slurry (Sotres et al. 2016). An anodic biofilm collected from a MFC fed with synthetic wastewater was used as inoculum. The pyrosequencing results showed that, as in the initial inoculum, *Proteobacteria*, *Bacteroidetes*, and *Firmicutes* were the most abundant phyla. However, the anodic biofilm presented higher percentage of operational taxonomic units (OTUs) shared with pig manure that was fed as substrate, than with the initial inoculum. This demonstrated the important role of substrate with respect to the microbial community developed in the anode. *Flavobacteriaceae* and *Chitinophagaceae* assigned to the *Bacteroidetes* phylum and *Comamonadaceae* and *Nitrosomonadaceae* assigned to the *Proteobacteria* phylum were the four predominant families identified. *Geobacter*, which contains well-known EAB, was not detected in the present study. Furthermore, several archaea were detected in the anodic microbial community, showing the competition between EAB and methanogens for the substrate. The archaea community was dominated by *Methanosarcinales* with a significantly lower percentage of *Methanomicrobiales* (Sotres et al. 2016). In the presence of high TAN concentration, *Methanosarcinales* have been reported to compete for electrons by establishing syntrophic interactions with acetate oxidizing microorganisms to produce methane (Schnürer and Nordberg 2008).

In another study, Barbosa et al. investigated the bacterial community changes in a MFC operated on human urine (Barbosa et al. 2017). The pyrosequencing results showed a process of enrichment and selection of the community. In comparison with the initial anaerobic inoculum, *Firmicutes* phylum was largely enriched in the anodic communities whereas the *Proteobacteria* phylum was reduced. *Paenibacillus*, *Clostridium*, *Atopostipes*, and *Tissierella* were the four main genera identified assigned to the Firmicutes phylum. *Paracoccus*, *Desulfobulbus*, and *Pseudomonas* were the three dominant genera assigned to the *Proteobacteria* phylum. The authors observed that the growth of fermentative bacteria (e.g., *Tissierella*), that degrade complex organics into acetate, seemed to play an important role for a stable current generation. Similarly to the previous work, bacteria belonging to the *Geobacter* genus were not detected in the developed community. Also, the diversion of electrons, due to the substrate consumption via other competing metabolic pathways was observed in this work. The authors identified the presence of *Methanomicrobiales*, a hydrogenotrophic methanogen, in the anodic biofilm (Barbosa et al. 2017).

A growing number of EAB for wastewater streams high in TAN have been discovered over the past years. The study of the interactions between EAB and non-EAB is crucial, especially in wastewater streams like urine that are rich in complex organics (Parameswaran et al. 2009). The co-existence of fermentative bacteria, acetogenic bacteria, methanogenic archaea, and EAB has been reported for BES using complex substrates (Jung and Regan 2007; Yang et al. 2012; Wang et al. 2016). Better understanding of these interactions could be useful to optimize BES operation and to ensure that most of the (complex) COD will be available for current generation at the bioanode.

## Biological aspects: ammonia toxicity

It is widely accepted that a high TAN concentration is considered toxic for microorganisms involved in anaerobic digestion (Yenigün and Demirel 2013). Free ammonia nitrogen (FAN, or NH_3_) in solution can easily penetrate microbial cells, disturbing the pH balance and inhibiting the enzymatic activity (Procházka et al. 2012). NH_3_ is proposed as the main form of TAN responsible for the inhibition of biological processes, rather than NH_4_^+^ (Nam et al. 2010). The concentration of FAN in solution is dependent on TAN concentration, pH, and temperature. At high pH (> pKa 9.25), most of the TAN is in the form of NH_3_ (Lide 1994). This toxicity can also reduce the activity of microorganisms in BESs and might therefore reduce current generation. However, the effect of TAN on the microorganisms involved in BESs is still unclear. There is no consensus about the threshold in which TAN (or NH_3_, depending on the study) is toxic or inhibitory in BESs. This threshold varies depending on the system operation, pH, conductivity, and acclimation period tested (Table 3). Additionally, the toxicity is often presented as a function of the influent TAN concentration, whereas the TAN concentration in the anolyte of a well-operated BES for TAN recovery will be considerably lower.

**Table 3:** Evaluation of important characteristics of ammonium/ammonia toxicity in recent literature focusing on (bio)electrochemical systems (BES); the main electron donor (substrate); the specific type (type) of BES (e.g., MFC, MEC); the mode of operation (mode), i.e., continuous (c) or batch (b); the reported anolyte pH; the reported anolyte conductivity; and the reported maximum current density (j, A m^−2^) with the respective maximum influent TAN concentration without inhibition

Substrate	Type	Mode	pH	Conductivity (mS cm^−1^)	Acclimation period	Maximum j (A m^−2^)	Maximum influent TAN without inhibition (g L^−1^)	Reference
Acetate	MEC	c	6.7 ± 0.1^a^	n.r.	Stepwise, from 1 to 5.5 g TAN L^−1^ (26 days)	5.3^c^	5	Clauwaert et al. (2008)
Acetate	MFC	b	7^b^	11.2^b^	Stepwise, from 0.08 to 4 g TAN L^−1^ (8 steps)	(4.2)^d^	0.5	Nam et al. (2010)
Acetate	MFC	c	7^b^	34.6^b^	Stepwise, from 0.08 to 10 g TAN L^−1^ (11 steps of 4–5 days each)	(6.1)^d^	3.5	Kim et al. (2011)
Acetate	MFC	c	< 7.1^a^	n.r.	Stepwise, from 0.07 to 4 g TAN L^−1^ (40 days)	6.0	4	Kuntke et al. (2011)
Acetate	MFC	c	6.8^a^	37.2^b^	Stepwise, from 0.07 to 4 g TAN L^−1^ (41 days)	0.5	4	Kuntke et al. (2012)
Urine	MFC	c	8.85^a^	35.0^b^	Stepwise, from 0.07 to 4 g TAN L^−1^ (acetate solution, 76 days)	2.7	4.05	Kuntke (2013)
Acetate	MFC	b	6–7^a^	n.r.	Stepwise, from 0.1 to 4 g TAN L^−1^ (high substrate concentration fed with high frequency)	(1.9)^e^	4	Tice and Kim (2014)
Acetate	MFC	b	6–7^a^	n.r.	Stepwise, from 0.1 to 4 g TAN L^−1^ (low substrate concentration fed with high frequency)	(2.0)^e^	3	Tice and Kim (2014)
Acetate	MFC	b	6–7^a^	n.r.	Stepwise, from 0.1 to 4 g TAN L^−1^ (high substrate concentration fed with low frequency)	(~ 1.3)^e^	2.5	Tice and Kim (2014)
Acetate	MFC	n.r.	8.5^b^	35.6^b^	Stepwise, from 0.1 to 6 g TAN L^−1^	(1.3)^f^	4	Lin et al. (2016)
Acetate	MFC	b	~ 8^a^	~ 15^b^	Stepwise, from 0.08 to 7.9 g TAN L^−1^ (urea)	(3.2)^e^	3.94	Wang et al. (2017)
Acetate	MFC	b	~ 6.5^a^	~ 55^b^	Stepwise, from 0.08 to 7.87 g TAN L^−1^	(2.3)^e^	5.25	Wang et al. (2017)
Acetate	MEC	c	7.1–7.45^a^	19.5 ± 0.5^b^	Stepwise (16 weeks)	37.7	5.88	Ledezma et al. (2017)
Acetate	MEC	c	7.0–8.1^a^	n.r.	Stepwise, from 0.2 to 4.4 g L^−1^	8.2	2.2	Mahmoud et al. (2017)

*n.r.* not reported

^a^Anodic effluent

^b^Anodic influent

^c^Based on cathode surface area

^d^Power density (W m^−3^)

^e^Power density (W m^−2^)

^f^Calculated power density (W m^−3^)

In one study, it was found that TAN inhibition depended on the substrate concentration and feed frequency (Tice and Kim 2014). Three conditions were tested in MFCs: high substrate concentration at high-frequency feed (2 g L^−1^ acetate every 2 days), low substrate concentration at high-frequency feed (0.67 g L^−1^ acetate every 2 days), and high substrate concentration at low-frequency feed (2 g L^−1^ acetate every 6 days). MFCs could withstand a higher concentration of TAN at high substrate concentration and high-frequency feed compared to either lower concentration or lower feed frequency (Table 3).

In addition, the potential inhibiting effect of TAN concentration and solution pH on current generation in BESs has been studied. Concentrations of 0.5 g L^−1^ of TAN at neutral pH have been reported to inhibit the power generation of a single-chambered MFC in batch mode (Nam et al. 2010). On the other hand, Clauwaert et al. (2008) did not find a negative effect in the bioanode performance of a double-chambered MEC treating synthetic wastewater up to a concentration of 5 g L^−1^ (at an anolyte pH of around 6.7) (Clauwaert et al. 2008). At a concentration of 5.5 g L^−1^, however, the current production was negatively affected. Similarly, in a study from Kuntke et al. (2011), synthetic urine at high TAN concentration (up to 4 g L^−1^) was fed to a double-chambered MFC and no negative effect was identified (Kuntke et al. 2011). They hypothesize that the reason for the lack of inhibition might be, among others, that the solution pH was lower than 7.1, resulting in very low FAN concentrations. In their following study, Kuntke et al. (2012) fed both synthetic and real urine to an MFC, with TAN concentrations of up to 4.05 g L^−1^ (Kuntke et al. 2012). Synthetic wastewater had a pH of 6.8–7, while urine had a pH of 8.85. Even though the urine had a higher pH compared to the synthetic wastewater, no negative effects on the performance of any of the MFCs were found. Similarly, Wang and co-workers found no power generation inhibition at a TAN concentration up to 3.9 g L^−1^ using a urea solution (Wang et al. 2017). In this study, the effluent had a pH value of ≈ 8. Another recent study also showed no inhibition while working with synthetic urine at TAN concentrations as high as 5.88 g L^−1^, at an anolyte pH in the range of 7.1–7.45 (Ledezma et al. 2017).

Furthermore, Lin and co-workers studied the effect of TAN concentrations (0.1 to 6.0 g L^−1^) at different initial pH values using synthetic media (Lin et al. 2016). The authors found a maximum power generation at a concentration of 4 g L^−1^, after which the current decreased with the increase of TAN. The authors hypothesized that the increase of power generation with the increase of TAN concentration up to 4 g L^−1^ might have been a result of the increase in conductivity (from 5.2 to 35.6 mS cm^−1^). Different pH values up to 9.5 were tested for a TAN concentration of 4 g L^−1^. The maximum power generation was obtained at a pH of 8.5, after which a severe inhibition was observed (pH 9.5). This inhibition was possibly due to the increase in FAN concentration in solution because of the pH increase.

On the contrary, Mahmoud and co-workers (2017) stated that EAB were resistant to relatively high FAN concentration, but sensitive to high TAN concentration (Mahmoud et al. 2017).The authors observed that even at a pH of 7.35, the EAB were sensitive to TAN concentrations > 2.2 g L^−1^. On the other hand, at 2.2 g TAN L^−1^, the current density increased up to a pH of 8.1, which corresponded to the highest FAN concentration tested (0.2 g FAN L^−1^). This FAN concentration, however, is very low compared to other BES studies (such as an anolyte concentration of 1.15 g FAN L^−1^ (Kuntke et al. 2012)).

Some studies reported that mixed cultures can be acclimated to TAN (Clauwaert et al. 2008; Kuntke et al. 2011; Kim et al. 2011; Kuntke et al. 2012; Ledezma et al. 2017). These studies claim that TAN inhibition can be overcome to a certain extent once the microbial community has been gradually adapted to high concentrations of TAN (stepwise). However, one of the studies which did follow a stepwise increase in TAN concentration still found inhibition at influent concentrations as low as 0.5 g L^−1^ (Nam et al. 2010). Mahmoud and co-workers suggested that these discrepancies are possibly caused by factors such as the diffusion of oxygen from the cathode to the anode chamber and the use of a cation-exchange membrane that allows the transport of NH_4_^+^ to the cathode (Mahmoud et al. 2017). Both factors can lead to the loss of TAN (either through nitrification or transport to the cathode) and result in a lower concentration of TAN in the anolyte, which might diminish the TAN inhibition. Nevertheless, there are studies mentioned in Table 3 that, even taking into account the TAN removals or losses, withstood to higher TAN concentrations in the anolyte than the 2.2 g L^−1^ reported by Mahmoud et al. (2017) (Clauwaert et al. 2008; Kim et al. 2011; Kuntke et al. 2012; Tice and Kim 2014; Ledezma et al. 2017).

Alternatively, ionic or osmotic stress has been mentioned as the cause of inhibition at high TAN concentrations. Müller et al. (2006) studied the effect of TAN on different model bacteria: *Corynebacterium glutamicum*, *Escherichia coli*, and *Bacillus subtilis* (Müller et al. 2006). They demonstrated that for *C. glutamicum*, which has been shown to be part of the microbial community in MFCs treating urine (Barbosa et al. 2017), there was no inhibition up to concentrations of 0.5 M TAN (≈ 9 g L^−1^). At 1 M, they observed a slight effect on growth and at 2 M, they saw a clear inhibition response (lag phase and decreased growth rate). However, the inhibition response was the same when (NH_4_)_2_SO_4_ was switched for Na_2_SO_4_. The same held for *E. coli* and *B. subtilis,* which showed impairment in growth starting from a concentration of 0.75 M TAN (≈ 13.5 g L^−1^), but similar response was observed when using sodium. They concluded then that the growth retardation for these three bacteria was not due to specific toxicity of TAN, but rather ionic or osmotic stress (Müller et al. 2006). Nam et al. (2010) and Kim et al. (2011) also conducted experiments to distinguish TAN inhibition from osmotic stress inhibition by replacing the NH_4_^+^ for K^+^. Both studies report a decline in power output with higher conductivities due to osmotic stress (Nam et al. 2010; Kim et al. 2011). However, Nam et al. (2010) argue that the inhibition due to TAN itself is higher than the one experienced by osmotic stress, whereas Kim et al. (2011) indicate a stronger inhibitory effect by osmotic stress from K^+^ than from TAN.

## Conclusions and perspectives

Several technologies for the recovery of TAN from wastewater streams exist. However, these technologies often require dosing of chemicals and/or are energy intensive. In recent years, (B)ES have shown promise as an energy-efficient alternative for the recovery of TAN. (B)ES offer the possibility to concentrate the TAN and can be integrated with conventional recovery concepts, such as ammonia (membrane) stripping and precipitation (struvite or ammonium bicarbonate). The decision of applying BES or ES for the most optimal treatment concept depends on the characteristics of the wastewater (biodegradability and COD/N ratio) and required rates which determine reactor size and treatment capacity.

As shown in this work, the L_N_ is a crucial parameter to steer TAN recovery and allows to compare performance of different types of (B)ES. In BESs, biodegradability of the substrate is important to have sufficient electrons for TAN recovery. In the field of BES in relation to high TAN levels, the sensitivity of EAB towards (ammonia) toxicity is still not well understood, with no clear outcomes, and further dedicated research is necessary.

For the implementation of TAN recovery by bioelectrochemical technologies from wastewater streams, the amount of (bioelectrochemically) biodegradable COD in relation to TAN needs to be examined. Systems need to be operated at the right conditions: TAN load and current have to be tuned (L_N_), depending on the aim of the treatment. If biodegradable COD/TAN ratios are not suitable, pretreatment could be used to either partly remove TAN, or increase bioavailable COD (degradation of complex organics). Furthermore, a posttreatment might be required to polish effluent in terms of COD in order to meet wastewater treatment standards. Finally, these technologies will need to be demonstrated at larger scale to show their true potential.
